# Evaluation of standard-of-care intravitreal aflibercept treatment practices in patients with diabetic macular oedema in the UK: DRAKO study outcomes

**DOI:** 10.1038/s41433-022-02367-x

**Published:** 2023-01-18

**Authors:** Sobha Sivaprasad, Faruque Ghanchi, Simon P. Kelly, Ajay Kotagiri, James Talks, Peter Scanlon, Hellen McGoey, Andrew Nolan, Moneeb Saddiq, Jackie Napier, Peter Morgan-Warren

**Affiliations:** 1 National Institute for Health Research, Moorfields Biomedical Research Centre, London, UK; 2 Bradford Teaching Hospitals NHS Foundation Trust, Bradford, UK; 3 Bolton Hospital NHS Foundation Trust, Bolton, UK; 4 South Tyneside and Sunderland NHS Foundation Trust, Sunderland, UK; 5 Newcastle Upon Tyne Hospitals NHS Foundation Trust, Newcastle Upon Tyne, UK; 6 Gloucestershire Hospitals NHS Foundation Trust, Cheltenham, UK; 7 Bayer Plc, Reading, UK; 8 Ipsen UK Ltd, Slough, UK; 9 O4 Research Limited, Belfast, UK

**Keywords:** Health care, Retinal diseases

## Abstract

**Background/Objectives:**

DRAKO (NCT02850263) was a 24-month, prospective, non-interventional, multi-centre cohort study enrolling patients with diabetic macular oedema (DMO) including central involvement. The study evaluated UK standard-of-care intravitreal aflibercept (IVT-AFL) treatment. This analysis describes the treatment pathway and service provision for the anti–vascular endothelial growth factor (VEGF) treatment-naïve (C1) and non-naïve patients (C2) who received prior anti-VEGF treatment for DMO other than IVT-AFL.

**Methods:**

Mean changes in best-corrected visual acuity and central subfield thickness were measured and stratified by baseline factors, including ethnicity and administration of five initial monthly injections within predefined windows. Clinic visits were classified as treatment only (T1), monitoring assessment only (T2), combined visits (T3) or post-injection visits with no treatment or assessment (T4).

**Results:**

Median time from decision to treat to treatment was 6 days. As a percentage of total visits, T1, T2, T3 and T4 were 7%, 42%, 48% and 3% for C1 and 11%, 39%, 48% and 2% for C2. Most IVT-AFL injections were administered by healthcare professionals (HCPs) other than doctors (C1, 57.4%; C2, 58.5%). The percentage of treatments associated with a procedure-related adverse event where at least 75% of injections were completed by the same injector role were similar for doctors and other HCPs (C1, 1.1% and 0.8%; C2, 0.7%, and 1.0%).

**Conclusions:**

Results indicate that upon DMO diagnosis, patients were treated promptly, and most visits were combined (treatment and assessment) or monitoring only. Most IVT-AFL was administered by non-physicians with a similar treatment-related safety profile as IVT-AFL administered by physicians.

## Introduction

Diabetes continues to increase in worldwide prevalence, and it is projected that 783.2 million adults between the ages of 20 and 79 will be affected by 2045 [1].

Diabetes management and its associated complications is a growing healthcare problem, with treatment estimated to cost around 10% of the UK’s entire National Health Service (NHS) budget, rising to 17% by 2035 [2].

In recent years, the UK government has launched various initiatives [3, 4] to reduce the prevalence of type 2 diabetes (T2D) and improve monitoring of diabetes. It is reported that there are 4.9 million people living with diabetes in the UK and a further 13.6 million at increased risk of developing T2D [5].

Diabetic retinopathy (DR) is the most common microvascular complication of diabetes [6], resulting from damage to retinal blood vessels. Diabetic macular oedema (DMO) is a manifestation of DR which can occur at any stage of retinopathy and is a primary cause of visual acuity (VA) loss in patients with diabetes [7, 8]. It is estimated that one in four patients with diabetes will develop DMO in their lifetime [9–11].

Since 2003, the UK has gradually implemented a national diabetic retinopathy screening programme [12], enabling early detection of disease complications in patients who are oftentimes asymptomatic [13].

Coincidentally, the last decade has seen the emergence of anti-vascular endothelial growth factor (anti-VEGF) treatments for DMO, improving outcomes for many patients.

Whilst these developments have been beneficial in DMO management, they have resulted in increased demands on healthcare services as patients are diagnosed earlier and clinicians utilise treatments, including intravitreal agents, to minimise vision loss.

The long-term focus on prevention of DMO is essential. However, management of this condition will remain a significant burden on healthcare systems for the foreseeable future. To improve services, stakeholders are seeking ways to improve service efficiency, including reducing duration and frequency of clinic visits and increasing multi-disciplinary team working.

Although the summary of product characteristics (SmPC) has now changed [14], when the first anti-VEGF for visual impairment secondary to DMO (ranibizumab) was originally licensed in the UK, monthly treatment was required until maximum attainable VA was achieved. Patients were then monitored monthly with further injections delivered as required in response to vision loss [15]. Such a regimen is effective, but continuous monthly visits to hospital are a significant burden for patients, caregivers and the health service. Intravitreal aflibercept (IVT-AFL) is an innovative anti-angiogenic treatment that was the first to offer an alternative to monthly treatment and/or monitoring for visual impairment due to DMO: a proactive regimen involving five initial monthly injections followed by every-other-month dosing with no mandatory monitoring between injections. After the first 12 months of treatment, the aflibercept treatment interval may be extended based on visual and anatomic outcomes [16]. DRAKO represents the first UK-based prospective, non-interventional study to assess standard of care IVT-AFL treatment in DMO patients across a wide range of centres.

The primary objectives of this study were to assess the mean changes from baseline in best-corrected visual acuity (BCVA) and central subfield thickness (CST) [17]. Here, we assess local follow-up procedures and resource management, including the numbers and types of patient visits, diagnostic assessments, timelines from diabetes and DMO diagnosis to treatment, management of bilateral DMO involvement, and healthcare professionals (HCPs) involved in administration of intravitreal injections within centres.

By reporting these endpoints, this manuscript aims to describe current UK practice and inform best practice guidance.

## Materials and methods

### Study design

The study design and methods have been published previously [17]. The principal study details are summarised here. DRAKO (NCT02850263) was a prospective, observational, multi-centre, non-comparative cohort study which evaluated the effectiveness of IVT-AFL for the treatment of DMO within UK routine clinical practice in 35 NHS centres. The study was approved by the Northwest Liverpool East Research Ethics Committee (16/NW/0238) and conducted in accordance with the Declaration of Helsinki. All participants provided written informed consent.

### Patients

DRAKO enrolled adult patients with a confirmed diagnosis of DMO with central involvement, into either the anti-VEGF treatment-naïve (*N* = 507) or anti-VEGF non-treatment-naïve (*N* = 241) cohort. Patients in the non-treatment-naïve cohort had not received anti-VEGF treatment 28 days prior to baseline and had not previously been treated with IVT-AFL. The study was primarily focused on outcomes for the study eye, defined as the eye with worse baseline VA in patients with bilateral DMO. However, to evaluate the DMO treatment service provision in the UK, fellow eye data were also captured. Patients enrolled in the study had to meet all previously published eligibility criteria [17], including confirmation that the investigator’s decision to administer IVT-AFL for DMO treatment was made prior to, and independent of, study involvement. Following removal of the requirement for patients to present with CST ≥ 400 µm at baseline by protocol amendment in February 2017, patients were enrolled irrespective of the baseline BCVA and CST. Patients were treated throughout the study as per local standard of care IVT-AFL treatment protocol for DMO. All visits, treatments, and monitoring assessments conducted throughout the 2-year follow-up period were collected alongside key study outcomes.

### Outcome measures

Primary study objectives were; mean visual change from baseline in Early Treatment Diabetic Retinopathy Study (ETDRS) letters, measured by BCVA with refraction, and mean change in CST as determined by spectral domain optical coherence tomography (SD-OCT) at month 12 (M12) for both cohorts. Results for the primary outcomes were reported previously [17].

Secondary objectives assessed the patient treatment pathway and service provision throughout the 2-year follow-up period for each cohort. Exploratory analysis further evaluated the primary outcomes by ethnicity and associated reported adverse events by IVT-AFL injection administrator.

### Statistical analysis

Two populations were defined for each cohort at M12 and Month 24 (M24), a ‘per protocol window’ (PPW) population including patients with BCVA or CST data available at baseline and the nominated M12 or M24 visit, and a full analysis set (FAS) population, including patients with BCVA or CST available at baseline and at least one follow-up visit. Where required, missing BCVA or CST data were imputed using the last observation carried forward. All analysis outcomes reported herein are based on the PPW population.

Analysis methods were published previously [17]. Briefly, quantitative variables were summarised by descriptive statistics and categorical variables by frequency distributions and percentages.

Primary outcomes were stratified by: (1) ethnicity recorded for patients within the study database; (2) administration of five initial monthly IVT-AFL treatments within 25-to-38-day windows (loading dose).

Visits were classified by type: treatment only (where an IVT-AFL injection was administered), monitoring assessment only (where at least one disease monitoring assessment or vital sign reading was conducted), combined visits (where treatment and one or more monitoring assessments were conducted) or post-injection visits (visit conducted post-treatment where neither treatment was given, or a monitoring assessment occurred). Visit and assessment data pertain to the study eye only.

All safety events reported over the 2-year follow-up period were coded using the Medical Dictionary for Regulatory Activities. Analysis was conducted using the safety population for each cohort (all patients who provided written informed consent), summarised by investigator-defined causality and stratified by IVT-AFL administrator, either physician as per IVT-AFL SmPC [16] or non-physician injector (e.g., nurse/optometrist).

Analysis was performed using SAS® software, version 9.4 (SAS Institute Inc., Cary, NC, USA).

## Results

### Patient pathway

**Fig. 1:**
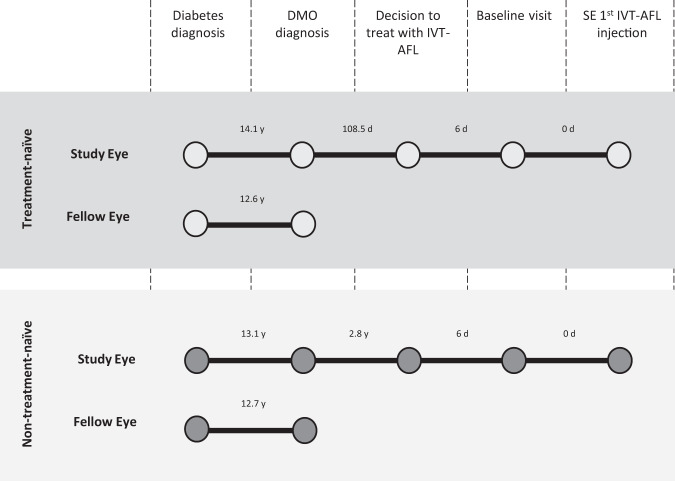
Patient care pathway, detailing the median time between events from diabetes diagnosis to first treatment with intravitreal aflibercept (IVT-AFL). Timeline data is outlined for both the treatment-naïve and non-treatment-naïve cohorts for the study eye (SE) and the fellow eye where applicable. d Days, DMO Diabetic macular oedema; y Years.

Key clinically relevant dates within the patient care pathway were assessed from diabetes diagnosis to the first post-baseline IVT-AFL injection (Fig. 1). The median time from diabetes diagnosis to DMO diagnosis within the study eye was 14.1 years for the treatment-naïve cohort and 13.1 years for the non–treatment-naïve cohort. The median time from DMO diagnosis to the decision to treat with IVT-AFL was longer in the non–treatment-naïve cohort (2.8 years) compared to the treatment-naïve cohort (108.5 days) and for most of these patients ranibizumab was administered as first-line therapy [17]. Notably, for both cohorts, the median times from the decision to treat with IVT-AFL to the baseline visit and subsequent IVT-AFL injection were 6 days and 0 days, respectively, indicating most patients were treated at their baseline visit. For patients presenting with fellow-eye involvement, the fellow eye was often the first eye to be diagnosed with DMO, yet had superior baseline BCVA to the study eye.

### Differences in functional and anatomical outcomes among ethnic subgroups

Mean change from baseline at M12 and M24 in BCVA and CST were evaluated based on patient-reported ethnicity (Supplementary Tables 1 and 2). Most patients were White (75.3% treatment-naïve and 62.7% non–treatment-naïve) and the proportion of Asian patients in the non–treatment-naïve cohort was more than double that reported in the treatment-naïve cohort (20.1% vs. 9.3% respectively). White patients had a mean baseline BCVA of 72.0 letters and 69.3 letters for the treatment-naïve and non–treatment-naïve cohorts, respectively. For ethnicity groups that were >5% proportion of the study population, the CST baseline measure was lowest in the Black population for the treatment-naïve (424.7 µm) and non–treatment-naïve cohorts (404.3 µm).

For the known ethnicity groups in the treatment-naïve cohort, the Asian population had the largest mean change from baseline in BCVA at M12 (4.3 letters) and the White population experienced a 2.2-letter gain (Supplementary Table 1). All ethnicity groups with >5% proportion of the study population experienced a reduction in BCVA at M24 compared to M12, although only the Black population fell below baseline (–0.7 letters). All ethnicity groups experienced an improvement in CST at M12, with mean changes from baseline of –129.7 µm and –117.7 µm for Asian and White groups, respectively. Predominantly, eyes continued to become drier in year 2 across most ethnicity groups.

In the non–treatment-naïve cohort, for the ethnicities of largest proportional representation, mean changes from baseline in BCVA outcomes were relatively stable at M12 (Supplementary Table 2). At M24, the mean change from baseline was –4.3 letters in Black patients, whereas mean BCVA remained stable (0.2 letters) in White patients. Improvements in CST were experienced across all ethnicity groups at M12 and M24, with the exception of the Hispanic population which included only a single patient. Overall, M12 and M24 mean changes from baseline outcomes varied depending on baselines measures.

### Treatment visits

In year 1, combined visits were predominant, with all patients attending at least one visit. A mean of 5.7 and 4.8 combined visits were recorded for treatment-naïve and non–treatment-naïve patients, respectively (Table 1). The mean number of treatment-only and monitoring assessment–only visits in year 1 was comparable for both cohorts.

**Table 1:** Summary of patient visits in year 1 (M12) and year 2 (M24) for the treatment-naïve and non-treatment-naïve patient cohorts. The number (*n*) and percentage of patients attending each visit category and the mean (SD) number of each visit category are defined.

	Treatment-naïve						Non-treatment-naïve					
	M12 (*n* = 388)			M24 (*n* = 326)			M12 (*n* = 169)			M24 (*n* = 135)		
**Visit Category**	***n*** **(%)**	**Mean (SD)**	**Total Visits (%)**	***n*** **(%)**	**Mean (SD)**	**Total Visits (%)**	***n*** **(%)**	**Mean (SD)**	**Total Visits (%)**	***n*** **(%)**	**Mean (SD)**	**Total Visits (%)**
**Treatment only**	74 (19.1)	3.8 (2.4)	281 (7.5)	75 (23.0)	5.2 (4.7)	390 (7.4)	53 (31.4)	3.8 (2.3)	201 (12.8)	39 (28.8)	5.8 (3.8)	226 (10.7)
**Monitoring assessment only**	344 (88.7)	3.4 (2.0)	1170 (31.3)	316 (96.9)	7.0 (3.9)	2212 (42.1)	148 (87.6)	3.5 (1.8)	518 (33.0)	132 (97.8)	6.2 (3.4)	818 (38.6)
**Combined**	388 (100.0)	5.7 (2.5)	2212 (59.2)	326 (100.0)	7.7 (4.0)	2510 (47.7)	169 (100.0)	4.8 (2.9)	811 (51.7)	135 (100.0)	7.6 (5.3)	1026 (48.4)
**Post-injection**	26 (6.7)	2.8 (2.2)	73 (2.0)	38 (11.7)	3.9 (4.0)	148 (2.8)	16 (9.5)	2.4 (1.9)	38 (2.4)	23 (17.0)	2.1 (1.4)	48 (2.3)

A treatment visit is defined as a visit where intravitreal aflibercept (IVT-AFL) is administered to the study eye.

A monitoring visit is defined as a visit where at least one of the following assessments is completed for the study eye: ocular assessments such as best-corrected visual acuity, spectral domain optical coherence tomography, non-refracted visual acuity; haemoglobin A1c or vital signs assessment.

A combined visit is defined as a visit where the study eye is treated with IVT-AFL and at least one monitoring assessment is conducted.

A post-injection monitoring visit is defined as a visit conducted following the first IVT-AFL treatment in the study eye, where neither treatment nor monitoring assessment(s) is conducted.

Over the 2-year follow-up period, combined visits were the most frequently attended visit type for both cohorts (mean of 7.7 visits and 7.6 visits for treatment-naïve and non–treatment-naïve cohorts, respectively) although the proportions were reduced in year 2, from 59% and 52% of total visits at M12 to 48% and 48% of visits at M24 for treatment-naïve and non–treatment-naïve cohorts, respectively. The proportions of monitoring assessment–only visits increased in year 2 for both cohorts (change of 11% and 6% in total from M12 to M24 for treatment-naïve and non–treatment-naïve cohorts, respectively). Fewer treatment-only visits were reported in year 2, and post-injection visits were conducted infrequently and accounted for less than 3% of visits for all patients.

When patients required bilateral treatment, fellow eye injections were administered during the same visit on most occasions across both cohorts (62.6% for treatment-naïve and 80.3% for non–treatment-naïve) (Supplementary Table 3).

### Disease monitoring assessments

**Fig. 2:**
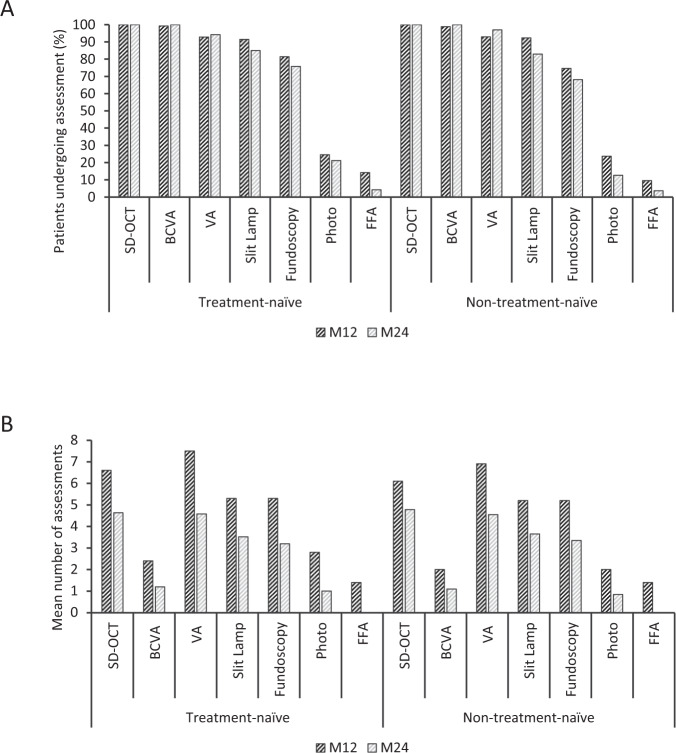
A summary of diabetic macular oedema disease monitoring assessments conducted during the study. **A** Shows the percentage of patients undergoing each assessment in year 1 (M12) and year 2 (M24) for treatment-naive and non-naive patients; **B** Shows the mean number of assessments per patient (accumulated figures are presented) in year 1 (M12) and year 2 (M24) for treatment-naive and non-naive patients. BCVA Best-corrected visual acuity, SD-OCT Spectral domain optical coherence tomography; VA Non-refracted visual acuity; Photo fundus photography; FFA fluorescein angiography, Slit lamp = slit lamp biomicroscopy; Ophthalmoscopy includes both direct and indirect assessment.

SD-OCT, BCVA, non-refracted VA and slit lamp biomicroscopy were conducted for more than 90% of patients in both cohorts in year 1 and 80% of patients in year 2 (Fig. 2).

Of the primary endpoint assessments, SD-OCT was conducted more frequently, with the mean frequency of both assessments reduced in year 2 (SD-OCT: treatment-naïve; year 1, 6.6, year 2, 4.6; non–treatment-naïve; year 1, 6.1, year 2, 4.8; BCVA: treatment-naïve; year 1, 2.4, year 2, 1.2; non–treatment-naïve; year 1, 2.0, year 2, 1.1). Frequency of assessments indicates a preference for non-refracted VA to assess functional disease outcomes. Fluorescein angiography was the least frequently conducted assessment with less than 1.5 mean assessments for both cohorts throughout the study period. Notably, the frequency of all assessments evaluated was reduced in year 2.

### Outcomes assessed by treatment pattern

The improved BCVA outcomes at M12 for treatment-naïve patients receiving five initial monthly injections (full loading dose) within window, in comparison to patients who did not receive these, was reported previously [17]. Further analysis for this cohort demonstrated that BCVA outcomes at the end of the follow-up period were marginally better in patients receiving the full loading dose compared to other patients (mean change from baseline of 1.4 letters compared to 0.4 letters, respectively); however, only one-third of patients received five loading injections (Table 2). Macular fluid dryness was comparable for those patients with or without full loading dose administration at M24 (mean CST change from baseline of –121.5 µm and –124.2 µm respectively).

**Table 2:** Mean change from baseline (BL) outcomes in best-corrected visual acuity (BCVA) and central subfield thickness (CST) for treatment-naïve and non-treatment-naïve cohorts by initial dosing. Those patients receiving initial intravitreal aflibercept (IVT-AFL) injections within window are defined as patients receiving five initial monthly injections within 28 days +10/-3 days (25-to-38-day window). All other eligible patients are included in the incomplete IVT-AFL initial injections subgroup.

			Treatment-naïve				Non-treatment-naïve			
			Initial IVT-AFL injections within window		Incomplete IVT-AFL initial injections		Initial IVT-AFL injections within window		Incomplete IVT-AFL initial injections	
			BL	Change from BL	BL	Change from BL	BL	Change from BL	BL	Change from BL
**BCVA (letters)**	**M12**	**Mean (SD)**	70.6 (12.1)	4.2 (10.9)	71.7 (12.0)	1.8 (12.6)	64.5 (14.2)	3.0 (14.8)	69.6 (13.6)	–0.3 (9.1)
		***n*** **(%)**	111 (28.6)	104 (26.8)	264 (68.0)	249 (64.2)	25 (14.8)	23 (13.6)	141 (83.4)	130 (76.9)
	**M24**	**Mean (SD)**	70.1 (12.6)	1.4 (15.6)	72.2 (12.3)	0.4 (11.1)	67.5 (9.1)	-3.1 (11.7)	69.9 (13.4)	0.2 (13.2)
		***n*** **(%)**	105 (32.2)	105 (32.2)	221 (67.8)	221 (67.8)	22 (16.3)	22 (16.3)	113 (83.7)	113 (83.7)
**CST (µm)**	**M12**	**Mean (SD)**	443.3 (79.9)	-108.5 (110.0)	451.1 (92.2)	-123.6 (118.9)	446.5 (107.7)	-95.2 (123.7)	414.4 (123.0)	-76.2 (140.2)
		***n*** **(%)**	117 (30.2)	116 (29.9)	271 (69.8)	270 (69.6)	26 (15.4)	25 (14.8)	143 (84.6)	141 (83.4)
	**M24**	**Mean (SD)**	451.0 (82.8)	-121.5 (115.7)	446.0 (74.6)	-124.2 (98.6)	447.0 (99.0)	-61.0 (112.9)	417.8 (121.0)	-97.5 (136.1)
		***n*** **(%)**	105 (32.2)	105 (32.2)	221 (67.8)	221 (67.8)	22 (16.3)	22 (16.3)	113 (83.7)	113 (83.7)

In non–treatment-naïve patients, a lower proportion received loading dose (approximately 15%) compared to treatment-naïve patients (Table 2). At M12, patients who received five loading injections experienced a mean gain from baseline of 3.0 letters, compared to a loss of 0.3 letters in other patients. Conversely, at M24 a 3.1 letter loss was observed for patients who received loading dose compared to a stable result for those who did not. These differences between the M12 and M24 outcomes for the non–treatment-naïve patients may be due to the low number of patients available in the subgroup for assessment. Mean changes in CST across the loading dose subgroups reported an improvement in CST outcome, however, outcomes were affected by baseline severity (Table 2).

### Adverse events reported by administrator

A total of 948 treatment-emergent adverse events (TEAEs) were reported, 711 in the treatment-naïve cohort and 237 in the non–treatment-naïve cohort. Investigator assessment indicated that 44 (6.2%) and 13 (5.5%) TEAEs had a reasonable causal relationship with the injection procedure, and 26 (3.7%) and 9 (3.8%) TEAEs had a reasonable causal relationship with IVT-AFL, in the treatment-naïve and non–treatment-naïve cohorts, respectively (Table 3). IVT-AFL injection administrator assessment indicated that the majority of injections were administered by HCPs other than physicians (Supplementary Table 4), primarily nurse administrators (treatment-naïve 55.0%, non–treatment-naïve 54.1%). For most patients, the administrator type varied throughout the 2-year follow-up, with only 6.2% treatment-naïve and 6.5% non–treatment-naïve patients receiving treatment by a physician throughout the study.

**Table 3:** Summary of treatment-emergent adverse events (TEAEs) with reasonable causal relationship with the injection procedure or intravitreal aflibercept (IVT-AFL) treatment in the treatment-naïve and non-treatment-naïve patient cohorts. TEAEs are stratified by injector, where the same category of injector has administered IVT-AFL injections throughout the study or where the same injector category has administered IVT-AFL 75% of all injections throughout the study. The Other HCP injector group includes all non-clinician healthcare professionals (HCPs), including nurses and optometrists. Analysis was conducted in the safety population.

	Injector role		Injection procedure-related TEAEs			IVT-AFL treatment-related TEAEs		
			TEAEs *n*	Patients *n* (%)	Injections by injector role associated with TEAE (%)	TEAEs *n*	Patients n (%)	Injections by injector role associated with TEAE (%)
**Treatment-naïve (*n*** = **507)**	**Any TEAE (*n*** = **711)**							
	All TEAEs/All injector roles		44	28 (5.5)	1.1	26	16 (3.2)	0.6
	Administrator of the last injection prior to TEAE	Doctor	27	14 (2.8)	1.6	14	8 (1.6)	0.8
		Other HCP	17	14 (2.8)	0.7	12	8 (1.6)	0.5
	Same injector role for 100% injections	Doctor	1	1 (0.2)	0.2	1	1 (0.2)	0.2
		Other HCP	3	3 (0.6)	0.4	1	1 (0.2)	0.1
	Same injector role for 75% injections	Doctor	11	6 (1.2)	1.1	7	5 (1.0)	0.7
		Other HCP	12	8 (1.6)	0.8	8	4 (0.8)	0.5
**Non-treatment-naïve (*n*** = **241)**	**Any TEAE (*n*** = **237)**							
	All TEAEs/All injector roles		13	10 (4.1)	0.8	9	6 (2.5)	0.5
	Administrator of the last injection prior to TEAE	Doctor	5	5 (2.1)	0.7	4	3 (1.3)	0.5
		Other HCP	8	5 (2.1)	0.7	5	3 (1.3)	0.4
	Same injector role for 100% injections	Doctor	2	2 (0.8)	0.7	1	1 (0.4)	0.3
		Other HCP	2	1 (0.4)	0.7	2	1 (0.4)	0.7
	Same injector role for 75% injections	Doctor	3	3 (1.2)	0.7	1	1 (0.4)	0.2
		Other HCP	6	3 (1.2)	1.0	6	3 (1.2)	1.0

The number of TEAEs and patients affected was consistent for both administrator types (physician vs. non-physician). Overall, in the treatment-naïve cohort, 1.6% of doctor-administered and 0.7% of other HCP-administered IVT-AFL injections were associated with a TEAE. For the non–treatment-naïve cohort, 0.7% of doctor-administered and 0.7% of other HCP-administered IVT-AFL injections were associated with a TEAE, respectively.

In patients who received at least 75% of injections from the same administrator group, the proportion of injections resulting in an injection-related TEAE was less than 1% in both the treatment-naïve (1.1% doctors, 0.8% other HCPs) and the non–treatment-naïve cohort (0.7% doctors, 1.0% other HCPs) (Table 3).

## Discussion

DRAKO previously reported that the mean baseline age for treatment-naïve patients was 62.9 years [17]. A recent study in the UK assessing over 400,000 patients found the mean age for T2D diagnosis is 60.4 years in men and 61.7 years in women [18]. With almost 90% of patients diagnosed with T2D, the DRAKO cohort indicates that the DMO population developed T2D at a much younger age. DMO diagnoses took 13.1 and 14.1 years for the treatment-naïve and non–treatment-naïve cohorts respectively, consistent with the consensus that duration of diabetes beyond 10 years is a significant risk factor for developing DMO [19].

DRAKO previously reported that 53.9% of treatment-naïve patients had a DMO diagnosis in the fellow eye at baseline [17] and in these patients the fellow eye was diagnosed a median of 1.5 years before the study eye.

Although the overall study cohort is broadly aligned with the UK population, the proportion of Asian and Black patients was higher, and for the non–treatment-naïve cohort, more than double that observed in the UK 2011 census data for England and Wales [20] whilst the proportion of White patients was lower (UK 2011 census: 7.5%, 3.3% and 86.0% vs. DRAKO: 20.3%, 7.5% and 64.3%, respectively). The higher proportion of Black patients was expected, as the prevalence of DR is known to be markedly higher in the Black population than the White population [21]. The Asian population in DRAKO was larger than expected; however, it is widely reported that the risk of developing diabetes is up to six times higher in South Asian patients than in White patients [22]. Additionally, DRAKO included several centres in areas of ethnic diversity with large South Asian populations.

Outcome findings were mixed, with Black patients achieving superior BCVA and CST outcomes to White patients at M12 for both cohorts; however, BCVA outcomes at M24 were poorer, albeit the sample sizes were small (22 and 21 patients at M12 and M24, respectively).

All participating centres confirmed that assessing BCVA was standard of care; however, in practice non-refracted VA assessments were routinely used. The resource required may have resulted in them being reserved for periodic use as a reference point rather than for day-to-day clinical decision making. The proportion of ‘treatment-only’ visits were relatively small, indicating that most clinics operated a combined ‘one-stop’ assessment and treatment clinic visit strategy. Where bilateral treatment was required, around 70% of fellow-eye injections were provided on the same day as the study eye. The figure was higher for the non–treatment-naïve cohort, perhaps reflecting greater clinical familiarity and more established treatment patterns in such patients. As well as fewer visits and assessments in year 2, the proportion of combined visits reduced and monitoring-only visits increased. This seems to suggest a greater focus on year 1 of treatment, perhaps reflecting findings from various randomised clinical trials [23, 24] that the largest gains are achieved in the first year. There were still significant numbers of visits in year 2; however, the majority were monitoring only visits, indicative of a change in approach. It is possible that a need to manage resources may have influenced a strategy of maintaining vision, rather than actively seeking further gains in year 2. It was previously reported that treatment-naïve patients who received the full initial five injection loading dose, as per SmPC, experienced a mean letter gain of 4.2, significantly higher than the overall mean gain of 2.5 letters [17]. This trend continued in the non–treatment-naïve cohort, where a mean letter gain of 3.0 (14.8) was observed in patients receiving the full loading dose compared to a loss of 0.3 (9.1) letters for those that did not, importantly reflecting the earlier findings that the five initial monthly injections should be completed to optimise outcomes (Table 2).

DRAKO reported that the majority of IVT-AFL injections were administered by HCPs other than doctors. Although the current SmPC references that “*Intravitreal injections must be carried out according to medical standards and applicable guidelines by a qualified physician experienced in administering intravitreal injections*” [16], the ‘off-label’ approach observed in DRAKO is now well established and widely adopted in the UK, with a strong evidence base [25–29]. In keeping with a growing trend within the NHS for professional development of HCPs [30, 31] to address the increasing levels of demand on NHS services, the Ophthalmic Common Clinical Competency Framework (OCCCF) [32] was launched in 2016 with the support of the Royal College of Ophthalmologists, Royal College of Nursing, College of Optometrists and Health Education England. The OCCCF curriculum provides standardised training (Ophthalmic Practitioner Training) to non-medical professionals working in ophthalmic secondary care across the UK, including training on the administration of intravitreal injections. The transformational aim is to enable increased clinic capacity, with other HCPs substituting for ophthalmologists in administering injections and ophthalmologists overseeing the service and focusing on other roles within the clinic. DRAKO did not observe any meaningful differences in patient safety outcomes based on the injector role, reflecting well-documented clinical efficacy and safety profiles for non-physician injectors [25–28] and supporting the rationale for current established UK practice.

DRAKO has some limitations often inherent in observational studies, such as inconsistent treatment administration, non-defined functional eligibility metrics and descriptive analyses, lacking statistical power for formal comparative testing. However, the prospective study design and wide range of contributing sites enabled treatment effects to be monitored across a diverse, UK-representative population. In summary, DRAKO demonstrates that the UK diabetic retinopathy screening programme is identifying patients with a high baseline BCVA, and they are being treated soon after diagnosis. Centres are managing resources by adopting ‘one-stop clinics’, aligning treatment visits for patients undergoing bilateral treatment, focusing more on treatment in year 1, and monitoring and maintenance in year 2. Non-refracted VA assessments are routinely used, and IVT-AFL is most often administered by non-physicians, with similar safety outcomes as physician-administered treatments. Overall, the high baseline BCVA was maintained at 24 months and anatomical outcomes continued to improve in year 2, although it is possible that wider adherence to the initial five monthly injections as recommended by the IVT-AFL SmPC could have improved outcomes further.

## Summary

### What was known before

- The effectiveness of intravitreal aflibercept (IVT-AFL) for treatment of diabetic macular oedema (DMO) patients has been demonstrated in several pivotal clinical trials (VIVID and VISTA) and non-UK focused observational studies (APOLLON), although such investigations primarily focused on patients with baseline visual acuity of <73 letters.
- Retrospective registry-based studies of anti-vascular endothelial growth factor (anti-VEGF) treatments have reported lower injection frequency and functional gains than randomised clinical trials.

### What this study adds

- DRAKO results for non-treatment-naïve eyes confirmed previous findings for treatment-naïve eyes that the five initial monthly injections optimised year 1 outcomes of IVT-AFL treatment of DMO patients in the UK.
- In UK standard of care IVT-AFL treatment of DMO patients, IVT-AFL was most often administered by healthcare professionals other than doctors, with safety outcomes similar to those treatments administered by doctors.
- DRAKO findings suggested that a greater focus is placed on treatment in year 1 in standard of care IVT-AFL treatment of DMO patients in the UK, with considerably fewer treatment visits and assessments in year 2 and a substantial increase in the number of monitoring only visits.

## Supplementary information

DRAKO Manuscript 3 Supplementary Tables 1-4

## Data Availability

The availability of the data underlying this publication will be determined later according to Bayer’s commitment to the EFPIA/PhRMA “Principles for responsible clinical trial data sharing”. This pertains to scope, time point and process of data access. As such, Bayer commits to sharing upon request from qualified scientific and medical researchers patient-level clinical trial data, study-level clinical trial data, and protocols from clinical trials in patients for medicines and indications approved in the United States (US) and European Union (EU) as necessary for conducting legitimate research. This applies to data on new medicines and indications that have been approved by the EU and US regulatory agencies on or after January 01, 2014. Interested researchers can use www.clinicalstudydatarequest.com to request access to anonymized patient-level data and supporting documents from clinical studies to conduct further research that can help advance medical science or improve patient care. Information on the Bayer criteria for listing studies and other relevant information is provided in the study sponsors section of the portal. Data access will be granted to anonymized patient-level data, protocols and clinical study reports after approval by an independent scientific review panel. Bayer is not involved in the decisions made by the independent review panel. Bayer will take all necessary measures to ensure that patient privacy is safeguarded.

## References

[1] International Diabetes Federation Diabetes Atlas ninth edition. 2019. https://diabetesatlas.org/upload/resources/2019/IDF_Atlas_9th_Edition_2019.pdf

[2] Hex N, Bartlett C, Wright D, Taylor M, Varley D (2012). Estimating the current and future costs of Type 1 and Type 2 diabetes in the UK, including direct health costs and indirect societal and productivity costs. Diabet Med.

[3] NHS Diabetes Prevention Programme. 2022. https://www.england.nhs.uk/diabetes/diabetes-prevention/

[4] National Institute for Health and Care Excellence. National strategy and policy to prevent type 2 diabetes. 2021. https://pathways.nice.org.uk/pathways/preventing-type-2-diabetes/national-strategy-and-policy-to-prevent-type-2-diabetes.

[5] Diabetes UK. Diabetes diagnoses double in the last 15 years. 2021. https://www.diabetes.org.uk/about_us/news/diabetes-diagnoses-doubled-prevalence-2021

[6] Fong DS, Aiello LP, Ferris FL, Klein R (2004). Diabetic retinopathy. Diabetes Care.

[7] Klein R, Klein BE, Moss SE, Davis MD, DeMets DL (1984). The Wisconsin epidemiologic study of diabetic retinopathy. III. Prevalence and risk of diabetic retinopathy when age at diagnosis is 30 or more years. Arch Ophthalmol.

[8] Moss SE, Klein R, Klein BE (1998). The 14-year incidence of visual loss in a diabetic population. Ophthalmology.

[9] Cohen SR, Gardner TW (2016). Diabetic retinopathy and diabetic macular edema. Dev Ophthalmol.

[10] Stefánsson E, Bek T, Porta M, Larsen N, Kristinsson JK, Agardh E (2000). Screening and prevention of diabetic blindness. Acta Ophthalmol Scand.

[11] Amoaku WM, Ghanchi F, Bailey C, Banerjee S, Banerjee S, Downey L (2020). Diabetic retinopathy and diabetic macular oedema pathways and management: UK Consensus Working Group. Eye.

[12] GOV.UK. Guidance: Diabetic eye screening: programme overview. 2021. https://www.gov.uk/guidance/diabetic-eye-screening-programme-overview.

[13] Brand CS (2012). Management of retinal vascular diseases: a patient-centric approach. Eye.

[14] European Medicines Agency. Lucentis Summary of Product Characteristics. 2022. https://www.ema.europa.eu/en/medicines/human/EPAR/lucentis.

[15] National Institute for Health and Care Excellence. Ranibizumab for treating diabetic macular oedema Technology appraisal guidance [TA274]. 2013. https://www.nice.org.uk/Guidance/TA274.

[16] European Medicines Agency. Eylea Summary of Product Characteristics. 2022. https://www.ema.europa.eu/en/medicines/human/EPAR/eylea.

[17] Sivaprasad S, Ghanchi F, Kelly SP, Kotagiri A, Talks J, Scanlon P (2022). Evaluation of standard of care intravitreal aflibercept treatment of diabetic macular oedema treatment-naive patients in the UK: DRAKO study 12-month outcomes. Eye.

[18] Martín-Merino E, Fortuny J, Rivero-Ferrer E, Lind M, Garcia-Rodriguez LA (2017). Risk factors for diabetic macular oedema in type 2 diabetes: A case-control study in a United Kingdom primary care setting. *Prim Care*. Diabetes.

[19] Varma R, Bressler NM, Doan QV, Gleeson M, Danese M, Bower JK (2014). Prevalence of and risk factors for diabetic macular edema in the United States. JAMA Ophthalmol.

[20] Office for National Statistics. 2011 Census. 2011. https://www.ons.gov.uk/census/2011census

[21] Sivaprasad S, Gupta B, Gulliford MC, Dodhia H, Mohamed M, Nagi D (2012). Ethnic variations in the prevalence of diabetic retinopathy in people with diabetes attending screening in the United Kingdom (DRIVE UK). PLoS One.

[22] The King’s Fund. The health of people from ethnic minority groups in England. 2021. https://www.kingsfund.org.uk/publications/health-people-ethnic-minority-groups-england#Diabetes.

[23] Nguyen QD, Brown DM, Marcus DM, Boyer DS, Patel S, Feiner L (2012). Ranibizumab for diabetic macular edema: results from 2 phase III randomized trials: RISE and RIDE. Ophthalmology.

[24] Brown DM, Schmidt-Erfurth U, Do DV, Holz FG, Boyer DS, Midena E (2015). Intravitreal aflibercept for diabetic macular edema: 100-week results from the VISTA and VIVID studies. Ophthalmology.

[25] DaCosta J, Hamilton R, Nago J, Mapani A, Kennedy E, Luckett T (2014). Implementation of a nurse-delivered intravitreal injection service. Eye (Lond).

[26] Simcock P, Kingett B, Mann N, Reddy V, Park J (2014). A safety audit of the first 10000 intravitreal ranibizumab injections performed by nurse practitioners. Eye (Lond).

[27] Michelotti MM, Abugreen S, Kelly SP, Morarji J, Myerscough D, Boddie T (2014). Transformational change: nurses substituting for ophthalmologists for intravitreal injections - a quality-improvement report. Clin Ophthalmol.

[28] Rasul A, Subhi Y, Sørensen TL, Munch IC (2016). Non-physician delivered intravitreal injection service is feasible and safe - a systematic review. Dan Med J.

[29] The Royal College of Ophthalmologists. Response from The Royal College of Ophthalmologists (RCOphth) to the HEE Strategic Framework Call for Evidence. 2021. https://www.rcophth.ac.uk/wp-content/uploads/2021/09/RCOphth-response-to-HEE-Strategic-Framework-Call-for-Evidence-6-Sept-final-1.pdf.

[30] Imison C, Castle-Clarke S, Watson, R Reshaping the workforce to deliver the care patients need. 2016. https://www.nuffieldtrust.org.uk/files/2017-01/reshaping-the-workforce-web-final.pdf

[31] National Health Service. Making the most of the skills in our teams. 2021. https://www.england.nhs.uk/ournhspeople/online-version/new-ways-of-working-and-delivering-care/making-the-most-of-the-skills-in-our-teams/.

[32] The Royal College of Ophthalmologists. New OCCCF curriculum launched to support professional development of the multi-disciplinary eye health team. 2019. https://www.rcophth.ac.uk/news-views/new-occcf-curriculum-launched-to-support-professional-development-of-the-multi-disciplinary-eye-health-team/

