# Supplementary material for: Evaluation of standard-of-care intravitreal aflibercept treatment practices in patients with diabetic macular oedema in the UK: DRAKO study outcomes
Source: Eye (Lond). 2023 Jan 18;37(12):2527–34. doi: 10.1038/s41433-022-02367-x (PMC10397211; doi:10.1038/s41433-022-02367-x)
Supplement: Supplementary file 1 — DRAKO Manuscript 3 Supplementary Tables 1-4 [file 41433_2022_2367_MOESM1_ESM.docx]

**Supplementary Table 1.** Mean change from baseline for M12 and M24 outcomes in best-corrected visual acuity (BCVA) and central subfield thickness (CST) for the treatment-naïve cohort by ethnicity.

|  |  | **M12**  **(n=388)** | | | | **M24**  **(n=326)** | | | |
| --- | --- | --- | --- | --- | --- | --- | --- | --- | --- |
|  |  | **Baseline** | | **Change from baseline** | | **Baseline** | | **Change from baseline** | |
|  | **Ethnicity** | **Mean (SD)** | **n**  **(%)** | **Mean (SD)** | **n**  **(%)** | **Mean (SD)** | **n**  **(%)** | **Mean (SD)** | **n**  **(%)** |
| **BCVA (letters)** | **Asian** | 66.8 (15.9) | 36  (9.3) | 4.3 (13.0) | 35  (9.0) | 65.9 (17.6) | 35  (10.7) | 2.3 (13.7) | 35  (10.7) |
|  | **Black** | 69.0 (12.8) | 25  (6.4) | 3.5 (14.1) | 22  (5.7) | 67.6 (13.7) | 21  (6.4) | -0.7 (19.0) | 21  (6.4) |
|  | **Hispanic** | 81.5 (3.5) | 2  (0.5) | -1.0 (1.4) | 2  (0.5) | 67.5 (23.3) | 2  (0.6) | 12.5 (16.3) | 2  (0.6) |
|  | **Mixed Race** | 73.0 (4.3) | 4  (1.0) | 1.0 (12.3) | 3  (0.8) | 73.7 (5.0) | 3  (0.9) | 10.3 (1.5) | 3  (0.9) |
|  | **Other** | 70.3 (3.2) | 3  (0.8) | 10.3 (7.2) | 3  (0.8) | 72.8 (5.5) | 4  (1.2) | 0.3 (6.9) | 4  (1.2) |
|  | **Unknown** | 73.2 (8.7) | 13  (3.4) | 1.3 (12.7) | 12  (3.1) | 74.3 (5.7) | 8  (2.5) | 0.0 (13.3) | 8  (2.5) |
|  | **White** | 72.0 (11.6) | 292  (75.3) | 2.2 (12.0) | 276  (71.1) | 72.5 (11.5) | 253  (77.6) | 0.4 (12.0) | 253  (77.6) |
| **CST (µm)** | **Asian** | 432.6 (158.9) | 38  (9.8) | -129.7 (171.2) | 38  (9.8) | 421.4 (92.5) | 35  (10.7) | -107.8 (90.2) | 35  (10.7) |
|  | **Black** | 424.7 (80.8) | 29  (7.5) | -125.4 (106.3) | 29  (7.5) | 430.4 (77.0) | 21  (6.4) | -130.1 (102.3) | 21  (6.4) |
|  | **Hispanic** | 497.5 (65.8) | 2  (0.5) | -99.0 (230.5) | 2  (0.5) | 438.0 (18.4) | 2  (0.6) | -86.0 (4.2) | 2  (0.6) |
|  | **Mixed Race** | 487.0 (110.7) | 4  (1.0) | -53.8 (227.3) | 4  (1.0) | 502.3 (130.2) | 3  (0.9) | -164.7 (163.0) | 3  (0.9) |
|  | **Other** | 459.3 (80.4) | 3  (0.8) | -175.3 (66.5) | 3  (0.8) | 451.5 (67.5) | 4  (1.2) | -143.5 (110.9) | 4  (1.2) |
|  | **Unknown** | 420.7 (55.9) | 14  (3.6) | -115.9 (92.6) | 14  (3.6) | 436.5 (39.1) | 8  (2.5) | -155.6 (64.2) | 8  (2.5) |
|  | **White** | 453.5 (77.3) | 298  (76.8) | -117.7 (108.1) | 296  (76.3) | 452.3 (75.2) | 253  (77.6) | -123.4 (107.3) | 253  (77.6) |

**Supplementary Table 2.** Mean change from baseline for M12 and M24 outcomes in best-corrected visual acuity (BCVA) and central subfield thickness (CST) for the non-treatment-naïve cohort by ethnicity.

|  |  | **M12**  **(n=169)** | | | | **M24**  **(n=135)** | | | |
| --- | --- | --- | --- | --- | --- | --- | --- | --- | --- |
|  |  | **Baseline** | | **Change from baseline** | | **Baseline** | | **Change from baseline** | |
|  | **Ethnicity** | **Mean (SD)** | **n**  **(%)** | **Mean (SD)** | **n**  **(%)** | **Mean (SD)** | **n**  **(%)** | **Mean (SD)** | **n**  **(%)** |
| **BCVA (letters)** | **Asian** | 69.1 (11.7) | 34  (20.1) | -1.6 (8.2) | 33  (19.5) | 69.7 (11.8) | 27  (20.0) | -0.9 (9.3) | 27  (20.0) |
|  | **Black** | 69.9 (8.8) | 13  (7.7) | 0.8 (11.8) | 11  (6.5) | 70.4 (7.3) | 10  (7.4) | -4.3 (13.0) | 10  (7.4) |
|  | **Hispanic** | 73.0  () | 1  (0.6) | 11.0 () | 1  (0.6) | 73.0  () | 1  (0.7) | 12.0  () | 1  (0.7) |
|  | **Mixed Race** | - | - | - | - | - | - | - | - |
|  | **Other** | 66.5 (15.3) | 4  (2.4) | 15.8 (19.6) | 4  (2.4) | 68.0 (16.1) | 4  (3.0) | 5.3 (7.8) | 4  (3.0) |
|  | **Unknown** | 59.6 (28.9) | 8  (4.7) | -0.1 (10.9) | 8  (4.7) | 68.4 (25.2) | 7  (5.2) | -2.9 (8.8) | 7  (5.2) |
|  | **White** | 69.3 (13.2) | 106  (62.7) | 0.0 (9.8) | 96  (56.8) | 69.5 (12.5) | 86  (63.7) | 0.2 (14.5) | 86  (63.7) |
| **CST (µm)** | **Asian** | 411.2 (82.1) | 34  (20.1) | -84.2 (120.3) | 33  (19.5) | 436.3 (70.0) | 27  (20.0) | -113.9 (117.6) | 27  (20.0) |
|  | **Black** | 404.3 (88.3) | 13  (7.7) | -92.8 (83.9) | 12  (7.1) | 395.5 (82.0) | 10  (7.4) | -60.0 (83.4) | 10  (7.4) |
|  | **Hispanic** | 295.0 () | 1  (0.6) | 18.0 () | 1  (0.6) | 295.0  () | 1  (0.7) | -16.0  () | 1  (0.7) |
|  | **Mixed Race** | - | - | - | - | - | - | - | - |
|  | **Other** | 461.5 (74.0) | 4  (2.4) | -112.5 (10.6) | 4  (2.4) | 473.8 (55.5) | 4  (3.0) | -103.5 (66.4) | 4  (3.0) |
|  | **Unknown** | 466.9 (112.6) | 8  (4.7) | -87.8 (117.4) | 8  (4.7) | 452.3 (117.8) | 7  (5.2) | -19.3 (143.1) | 7  (5.2) |
|  | **White** | 419.7 (135.8) | 109  (64.5) | -75.1 (151.9) | 108  (63.9) | 418.1 (134.3) | 86  (63.7) | -94.5 (143.2) | 86  (63.7) |

**Supplementary Table 3.** Summary of fellow eye treatments at the completion of the 2-year follow-up period for treatment-naïve and non-treatment-naïve cohorts. Study eye treatments in the same patient cohort populations have been included for reference.

|  | **Treatment-naïve (n=326)** | | | **Non-treatment-naïve (n=135)** | | |
| --- | --- | --- | --- | --- | --- | --- |
|  | **Patients n (%)** | **Number of injections**  **Mean (SD)** | **Total number of treatments** | **Patients n (%)** | **Number of injections Mean (SD)** | **Total number of treatments** |
| ***Study eye treatments*** | *326 (100)* | *8.9*  *(3.7)* | *2901* | *135 (100)* | *9.3*  *(4.4)* | *1256* |
| **Fellow eye treatments** | 124 (38.0) | 6.3  (3.9) | 781 | 73  (54.1) | 7.3  (4.4) | 533 |
| **Same day as study eye** | 97  (29.8) | 5.0  (3.6) | 485 | 67  (49.6) | 6.3  (4.2) | 422 |
| **Separate day to study eye** | 81  (24.8) | 3.7  (3.2) | 300 | 40  (29.6) | 2.8  (2.5) | 112 |

**Supplementary Table 4.** Number (%) of injections by intravitreal aflibercept (IVT-AFL) injector role for the treatment-naïve and non-treatment-naïve cohorts.

| **IVT-AFL injector role** | **Treatment-naïve**  **n (%)** | **Non-treatment-naïve**  **n (%)** |
| --- | --- | --- |
| **Doctors** | 1721 (41.1) | 755 (40.1) |
| **Nurse** | 2300 (55.0) | 1017 (54.1) |
| **HCP** | 99 (2.4) | 83 (4.4) |
| **Unknown** | 64 (1.5) | 26 (1.4) |
| **Total** | 4184 | 1881 |
